# The Gender-Specific Association between Age at First Drink and Later Alcohol Drinking Patterns in Korea

**DOI:** 10.1371/journal.pone.0090713

**Published:** 2014-03-04

**Authors:** Minsun Kang, Jae-Hyun Kim, Woo-Hyun Cho, Eun-Cheol Park

**Affiliations:** 1 Department of Public Health, Graduate School, Yonsei University, Seoul, Korea; 2 Institute of Health Services Research, Yonsei University, Seoul, Korea; 3 Eulji University, Daejeon, Korea; 4 Department of Preventive Medicine, Yonsei University College of Medicine, Seoul, Korea; Centre for Addiction and Mental Health, Canada

## Abstract

This study investigated the association between the age at first drink and later alcohol drinking patterns, and analyzed whether differences in the association exist among Korean adults according to gender. The subjects included 10,649 adults (5,405 men and 5,244 women) from the fourth Korean National Health and Nutrition Examination Survey between 2007 and 2009, which extracted the standard survey household by using the proportional systematic sampling method. Baseline individual characteristics, the age at first drink, and individual alcohol drinking patterns were obtained by specially trained interviewers or examiners. The association between the age at first drink and the adult alcohol drinking patterns was summarized with odds ratios and their confidence intervals obtained from multiple logistic regression analysis with sampling weights of KNHANES complex sample survey design. The results of this study show that age, co-habitation, occupation, smoking, and self-rated stress level were significantly related to the drinking patterns for men, whereas education, co-habitation, smoking, and self-rated stress level were significant factors for the drinking patterns of women. The association between the age at first drink and the adult alcohol consumption was significant for both genders and, interestingly, the alcohol drinking patterns were significantly differed by gender even after controlling for the individual characteristics. These results imply a need for gender-specific strategies to prevent hazardous alcohol consumption at a later time for Korean.

## Introduction

Alcohol use disorders are leading preventable causes of death and disability worldwide [1], [2]. Excessive alcohol consumption causes more than 4,500 deaths annually in Korea, and the alcohol-induced death rate was 9.1 per 100,000 individuals in 2010 [3]. Unhealthy alcohol use is prevalent among a large portion of Korean men, and the prevalence of alcohol dependence and alcohol abuse is much higher in Korea compared to other countries [4], [5].

Numerous studies have demonstrated a correlation between the age at first drink and alcohol use disorders later in life [5]–[8]. Therefore, delaying the onset of drinking behavior would be an important aim in alcohol use disorder prevention programs. However, in reality the adolescent drinking rate gradually increases with age, and female adolescent drinking rates approach those of males [8], [9]. These issues are recently of growing international concern.

Starting to drink at an early age may lead to prolonged neurobiological effects specific to the adolescent brain [10], including various cognitive and social problems [11] as well as binge drinking in adulthood. Indeed, there is much evidence converging to support this hypothesis. Significant associations between the age at first drink and later heavy use of alcohol have been found in several studies. Some authors have suggested a positive association between the age at first drink and alcohol consumption [12]–[14], frequency of drinking at young ages [15], alcohol abuse and dependence in adulthood [16], [17], frequency of heavy drinking [18], and consequent binge drinking in adulthood [19]. Additionally, several studies show significant relationships between the age at first drink and the risk for unintentional injury with the use of alcohol [20] and drugs [11], [21]. Based on these previous studies, it might seem that the age at first drink would be one of major factors related with later hazardous alcohol consumption, but most studies have not yet assessed for different gender-specific relationships. This study aimed to analyze the gender-specific association between the age at first drink and later alcohol drinking patterns after controlling for possible confounders which may influence alcohol consumption behaviors using the Korea National Health and Nutrition Examination Survey data.

## Materials and Methods

### Study Data

Data were obtained from the fourth Korean National Health and Nutrition Examination Survey (KNHANES IV) between 2007 and 2009. The KNHANES is a nationwide representative survey using a stratified and multistage probability sampling design for the selection of household units. The survey was conducted by the Korean Ministry of Health and Welfare to establish national health policy based on the evaluation of health risk factors and health indicators. The survey data were obtained by specially trained interviewers or examiners who were not provided with any prior information about the survey participants and all participants signed an informed consent form before the survey was conducted. More details on the research methodology used to gather and analyze the KNHANES are available from the KNHANES website.

Participants over the age of 18 years were identified for this study. A total of 10,649 individuals (5,405 men and 5,244 women) were included as our final study population after excluding persons who never consumed alcohol.

### Measures

Baseline individual characteristics regarding gender, age, household income, education level, co-habitation, residential region, occupation, smoking, and self-rated stress level were collected for all cases. Age was categorized into five groups (≤ 29, 30–39, 40–49, 50–59, and ≥60). Household income was classified into four groups: low, middle-low, middle-high, and high. Education level was categorized as follows: elementary school graduate and under, middle school graduate, high school graduate, and college graduate or post-graduate. Co-habitation referred to whether the individual lived with a spouse. With regard to residential region, participants were classified into living in Seoul, living in an urban area, and living in a rural area. Occupation was categorized as follows: white-collar jobs, blue-collar jobs, and unemployed (including student and housewife). Smoking status was categorized into nonsmoker with no past smoking experience, ex-smoker, and current smoker. Self-rated stress level was categorized as follows: very much, much, little, and almost none.

Self-reported questionnaires were used to assess each participant’s alcohol related behaviors. For the age at first drink, participants were asked the age at which they first started drinking, not counting tastes or sips, which was classified into eight categories (≤14, separately for each year from 15 through 20 years old, and ≥21). Three indicators of the adult alcohol drinking patterns were used: The Alcohol Use Disorder Identification Test (AUDIT) score, average drinking frequency in past year, and typical drinking quantity per drinking day in past year. AUDIT is a 10-item screening instrument developed by the World Health Organization for the early detection of persons with harmful alcohol consumption behavior [22]. The AUDIT summary score range is 0 to 30. In this study, AUDIT was classified into two categories (≥8 and <8) based on that a score of 8 or higher is usually considered as at-risk drinking level [22]. Average drinking frequency in past year was assessed with five categories(<1/month, 1/month, 2–4/month, 2–3/week, and ≥4/week) and typical drinking quantity per drinking day in past year was assessed with five categories(1 or 2 drinks, 3 or 4 drinks, 5 or 6 drinks, 7–9 drinks, and ≥10 drinks). In these questions, a ‘drink’ was defined as one serving of alcoholic beverages regardless of the kind including beer, whisky, or Soju which is Korean traditional liquor. One serving of these beverages contains 8–9 g alcohol, even though each drink has different volumes. In this study, we considered participants having ≥8 on the AUDIT, or reporting average drinking ≥2–3 times/week, or consuming more than 5–6 drinks/drinking day as individuals with at-risk drinking level based on previous studies [5], [22]. Therefore, average drinking frequency was classified into two groups (≥2–3/week and <2–3/week) and typical drinking quantity per drinking day was classified into two groups (≥5 or 6 drinks and < 5 or 6 drinks).

### Statistical analyses

Statistical analyses were performed using SAS version 9.2 (SAS Institute Inc, Cary, NC, USA) to take into account the complex sampling design with sampling weights of KNHANES and provide nationally representative estimates. Descriptive statistics including means, standard deviation, and frequency for estimates of the adult alcohol drinking patterns were calculated for men and women separately since there was no interaction found. Baseline characteristics of the study participants according to the alcohol drinking patterns were compared using the chi-square test. The gender-specific alcohol drinking patterns were compared using a two sample t-test for continuous variables and the chi-square test for categorical variables. The gender-specific effects of the age at first drink on adult alcohol drinking patterns were tested using the chi-square test. Multiple logistic regression analysis was used to find out the relationships between the age at first drink and the adult alcohol drinking patterns after adjusting for potential confounders. Odds ratios and 95% confidence intervals (CIs) were calculated to describe the association between the age at first drink and the drinking patterns for those with age 14 years and under at first drink and each age from 15 through 20 years relative to those with age 21 years or older at first drink, which was the reference group. The sample weights account for sampling variability and adjust the data for differential probability of selection of persons in KNHANES complex sample survey design.

## Results

### Characteristics of the study participants

The characteristics of 10,649 individuals based on the alcohol drinking pattern are shown in Table 1. There is no significant effect of residential region on the three indicators of the adult alcohol drinking patterns for both genders. Age, co-habitation, occupation, smoking, and self-rated stress level were significantly related to AUDIT, average drinking frequency and typical drinking quantity for men. Household income and education were significantly related to average drinking frequency and typical drinking quantity for men. Education, co-habitation, smoking, and self-rated stress level were significantly related to AUDIT, average drinking frequency and typical drinking quantity for women. There is no significant effect of household income on the women’s drinking patterns.

**Table 1:** Characteristics of the study population (number, %).

	Men (n = 5405)									
	AUDIT ≥ 8		AUDIT < 8	*p*-value	ADF ≥2–3/week	ADF <2–3/week	*p*-value	TDQ ≥ 5 or 6	TDQ < 5 or 6	*p*-value
Age										
≤ 29	471 (19.3)		330 (23.8)	<.0001	219 (13.1)	582 (26.9)	<.0001	603 (23.3)	198 (15.9)	<.0001
30–39	780 (26.5)		432 (24.6)		479 (23.2)	733 (27.8)		897 (27.8)	315 (21.3)	
40–49	858 (28.4)		372 (21.1)		627 (29.8)	603 (22.6)		897 (27.6)	333 (21.5)	
50–59	628 (16.7)		301 (15.5)		526 (20.3)	403 (13.2)		596 (15.1)	333 (18.8)	
≥ 60	644 (9.1)		589 (15.0)		672 (13.6)	561 (9.5)		445 (6.2)	788 (22.5)	
Household income										
Low	505 (11.3)		332 (11.3)	0.867	442 (12.6)	395 (10.4)	0.004	428 (10.0)	409 (14.2)	0.001
Middle-low	803 (23.0)		499 (23.8)		655 (25.3)	647 (21.8)		798 (23.0)	504 (24.0)	
Middle-high	989 (31.3)		589 (32.0)		702 (30.6)	876 (32.3)		1,048 (32.0)	530 (30.7)	
High	1,084 (34.3)		604 (32.9)		724 (31.6)	964 (35.5)		1,164 (35.0)	524 (31.1)	
Education										
≤ Elementary school	571 (11.4)		321 (9.6)	0.091	566 (15.7)	326 (6.9)	<.0001	429 (8.5)	463 (15.6)	<.0001
≤ Middle school	412 (10.6)		239 (9.3)		365 (13.1)	286 (7.8)		353 (9.4)	298 (11.7)	
≤ High school	1,320 (43.3)		782 (43.6)		920 (41.0)	1,182 (45.3)		1,445 (45.2)	657 (39.6)	
≥ College	1,078 (34.7)		682 (37.5)		672 (30.1)	1,088 (40.1)		1,211 (37.0)	549 (33.0)	
Co-habitation										
Married	2,615 (73.1)		1,537 (69.7)	0.002	2,071 (78.3)	2,081 (67.0)	<.0001	2,531 (69.6)	1,621 (76.8)	<.0001
Single	574 (22.1)		405 (26.9)		300 (16.5)	679 (29.4)		726 (26.1)	253 (19.0)	
Separated	88 (1.9)		50 (1.7)		74 (2.2)	64 (1.5)		79 (1.6)	59 (2.2)	
Divorced	104 (2.9)		32 (1.7)		78 (3.0)	58 (2.0)		102 (2.7)	34 (2.0)	
Residential region										
Capital city	536 (20.8)		382 (23.5)	0.137	390 (20.4)	528 (22.8)	0.074	590 (21.7)	328 (21.9)	0.802
Urban	1,588 (48.5)		941 (48.2)		1,140 (47.8)	1,389 (48.8)		1,646 (48.7)	883 (47.6)	
Rural	1,257 (30.7)		701 (28.4)		993 (31.8)	965 (28.4)		1,202 (29.6)	756 (30.6)	
Occupation										
White-collar	1,433 (49.0)		748 (43.8)	<.0001	924 (44.6)	1,257 (49.1)	<.0001	1,572 (50.6)	609 (39.5)	<.0001
Blue-collar	1,381 (37.5)		802 (37.1)		1,158 (42.4)	1,025 (33.5)		1,306 (35.5)	877 (41.5)	
None	567 (13.4)		474 (19.0)		441 (13.0)	600 (17.4)		560 (13.9)	481 (19.1)	
Smoking										
Nonsmoker	431 (12.9)		451 (23.0)	<.0001	268 (10.2)	614 (21.5)	<.0001	485 (14.2)	397 (21.8)	<.0001
Ex-smoker	1,118 (30.0)		807 (36.4)		882 (31.2)	1,043 (33.2)		1,081 (29.0)	844 (39.7)	
Current	1,832 (57.2)		766 (40.6)		1,373 (58.6)	1,225 (45.3)		1,872 (56.8)	726 (38.5)	
Self-rated stress										
Very much	183 (5.2)		73 (3.3)	0.002	130 (5.0)	126 (4.1)	0.018	172 (4.7)	84 (4.1)	0.012
Much	791 (24.5)		398 (21.4)		558 (23.7)	631 (23.1)		801 (24.0)	388 (22.0)	
Little	1,900 (57.6)		1,177 (59.8)		1,373 (56.0)	1,704 (60.3)		1,985 (58.8)	1,092 (57.7)	
Almost none	507 (12.7)		376 (15.5)		462 (15.3)	421 (12.5)		480 (12.6)	403 (16.3)	
	Women (n = 5244)									
	AUDIT ≥ 8		AUDIT < 8	*p*-value	ADF ≥2–3/week	ADF <2–3/week	*p*-value	TDQ ≥ 5 or 6	TDQ < 5 or 6	*p*-value
Age										
≤ 29	326 (42.6)		610 (20.2)	<.0001	134 (24.9)	802 (25.2)	0.8571	425 (46.0)	511 (18.3)	<.0001
30–39	273 (23.6)		1,064 (24.8)		210 (25.1)	1,127 (24.4)		320 (24.1)	1,017 (24.6)	
40–49	232 (21.0)		1,022 (26.6)		204 (26.7)	1,050 (25.1)		244 (18.8)	1,010 (27.5)	
50–59	115 (9.2)		724 (16.1)		115 (13.8)	724 (14.8)		119 (8.3)	720 (16.7)	
≥ 60	67 (3.5)		811 (12.3)		124 (9.6)	754 (10.5)		58 (2.7)	820 (12.9)	
Household income										
Low	155 (12.1)		687 (13.5)	0.0261	144 (14.7)	698 (12.9)	0.0694	168 (12.2)	674 (13.6)	0.3387
Middle-low	287 (29.4)		1,008 (24.2)		198 (26.5)	1,097 (25.2)		305 (27.4)	990 (24.7)	
Middle-high	303 (30.0)		1,214 (29.5)		239 (31.8)	1,278 (29.2)		355 (30.3)	1,162 (29.4)	
High	268 (28.5)		1,322 (32.7)		206 (26.9)	1,384 (32.7)		338 (30.2)	1,252 (32.3)	
Education										
≤ Elementary school	156 (11.0)		1,059 (19.0)	<.0001	192 (18.5)	1,023 (17.0)	<.0001	147 (9.4)	1,068 (19.9)	<.0001
≤ Middle school	121 (11.5)		459 (10.7)		99 (14.1)	481 (10.3)		101 (8.3)	479 (11.7)	
≤ High school	516 (53.3)		1,551 (39.6)		356 (48.0)	1,711 (41.6)		619 (54.4)	1,448 (38.7)	
≥ College	220 (24.2)		1,162 (30.6)		140 (19.5)	1,242 (31.0)		299 (28.0)	1,083 (29.6)	
Co-habitation										
Married	557 (50.1)		3,048 (70.5)	<.0001	513 (63.2)	3,092 (66.6)	0.0041	625 (49.3)	2,980 (71.6)	<.0001
Single	298 (37.5)		525 (16.8)		117 (20.3)	706 (21.5)		382 (39.9)	441 (15.2)	
Separated	85 (6.4)		529 (9.8)		102 (10.4)	512 (8.8)		79 (5.4)	535 (10.2)	
Divorced	73 (6.0)		129 (2.9)		55 (6.1)	147 (3.1)		80 (5.4)	122 (3.0)	
Residential region										
Capital city	196 (22.1)		704 (21.4)	0.8994	145 (22.6)	755 (21.3)	0.4427	223 (21.7)	677 (21.5)	0.8877
Urban	478 (48.8)	2,058 (49.7)			373 (50.8)	2,163 (49.3)		565 (48.8)	1,971 (49.8)	
Rural	339 (29.1)	1,469 (28.9)			269 (26.6)	1,539 (29.4)		378 (29.5)	1,430 (28.8)	
Occupation										
White-collar	476 (51.6)	1,486 (41.5)		<.0001	326 (45.6)	1,636 (43.4)	0.2737	549 (51.4)	1,413 (41.2)	<.0001
Blue-collar	174 (15.5)	886 (18.2)			172 (18.8)	888 (17.3)		174 (13.1)	886 (19.0)	
None	363 (32.9)	1,859 (40.3)			289 (35.7)	1,933 (39.3)		443 (35.5)	1,779 (39.8)	
Smoking										
Nonsmoker	688 (65.6)	3,819 (89.5)		<.0001	561 (67.3)	3,946 (87.4)	<.0001	809 (67.9)	3,698 (89.7)	<.0001
Ex-smoker	106 (11.9)	225 (6.0)			79 (12.3)	252 (6.3)		120 (11.3)	211 (5.9)	
Current	219(22.5)	187 (4.5)			147 (20.4)	259 (6.2)		237 (20.8)	169 (4.4)	
Self-rated stress										
Very much	86 (9.3)	212 (5.3)		<.0001	59 (8.9)	239 (5.7)	0.0089	88 (8.2)	210 (5.5)	<.0001
Much	348 (34.9)	1,055 (26.0)			234 (30.4)	1,169 (27.5)		385 (33.9)	1,018 (26.0)	
Little	494 (48.1)	2,444 (57.6)			415 (52.5)	2,523 (56.1)		597 (49.8)	2,341 (57.4)	
Almost none	85 (7.7)	520 (11.0)			79 (8.3)	526 (10.7)		96 (8.0)	509 (11.0)	

Abbreviations: AUDIT, Alcohol Use Disorder Identification Test; ADF, average drinking frequency in past year; TDQ, typical drinking quantity (drinks/drinking day).

*Note*: *p*-values are based on the chi-squared test for categorical variables.

### Alcohol drinking patterns and gender

Just over half (59%) of the respondents were men and 41% were women. The mean AUDIT score was significantly higher for the men than the women with a mean AUDIT of 10.9±7.1 for the men and 4.7±5.0 for the women respectively (Table 2). Among men, 43% reported average drinking ≥2–3 times/week in past year and 69% of men consumed more than 5–6 drinks/drinking day. However, only 16% of women reported average drinking ≥2–3 times/week in past year and 25% of women consumed more than 5–6 drinks/drinking day (Table 2).

**Table 2:** The association between alcohol drinking patterns and gender a.

	Overall		Men		Women		*p*-value b
n (%)	10,649	(100%)	5,405	(59%)	5,244	(41%)	-
Alcohol drinking patterns in adulthood							
AUDIT score	8.5		10.9	(7.1)	4.7	(5.0)	<.0001
ADF ≥ 2–3/week	3,310		2,523	(43%)	787	(16%)	<.0001
TDQ ≥ 5 or 6 drinks/drinking day	4,604		3,438	(69%)	1,166	(25%)	<.0001

Abbreviations: AUDIT, Alcohol Use Disorder Identification Test; ADF, average drinking frequency in past year; TDQ, typical drinking quantity.

a Data are expressed as the mean (SD) or n (%) by weighted number.

b *p*-values were calculated using a two sample t-test or chi-square test.

### Age at first drink

Table 3 showed inverse associations between the age at first drink and the adult alcohol drinking patterns for both genders. Among those men who first started drinking at age 14 or under, 71% had ≥8 on the AUDIT, 54% reported average drinking ≥2–3 times/week in past year and 73% consumed more than 5–6 drinks/drinking day. Among those men who first started drinking at age 21 or older, only 54% had ≥8 on the AUDIT, 49% reported average drinking ≥2–3 times/week in past year and 47% consumed more than 5–6 drinks/drinking day. In the group of women, 47% of those who started drinking at age 14 or under had ≥8 on the AUDIT, 22% reported average drinking ≥2–3 times/week in past year and 49% consumed more than 5–6 drinks/drinking day, whereas 14% of those who started drinking at age 21 or older had ≥8 on the AUDIT, 15% reported average drinking ≥2–3 times/week in past year and 14% consumed more than 5–6 drinks/drinking day (Table 3). The interactions of gender and the age at first drink were not statistically significant at 0.01 level on the AUDIT score (F_470_  = 2.18, P  = 0.0347), average drinking frequency (F_470_  = 1.42, P  = 0.1939), and typical dinking quantity per drinking day (F_470_  = 2.44, P = 0.0183).

**Table 3:** Alcohol drinking patterns in adult by the age at first drink and gender a.

Age at first drink (year)	Men				Women			
	Total	AUDIT ≥ 8 b	ADF ≥2–3/week b	TDQ ≥ 5 or 6 b	Total	AUDIT ≥ 8 b	ADF ≥2–3/week b	TDQ ≥ 5 or 6 b
≤14	350 (8%)	250 (71%)	189 (54%)	255 (73%)	108 (3%)	51 (47%)	24 (22%)	53 (49%)
15	359 (7%)	249 (69%)	189 (53%)	253 (70%)	127 (3%)	48 (38%)	24 (19%)	60 (47%)
16	428 (9%)	307 (72%)	212 (50%)	338 (79%)	144 (3%)	60 (42%)	30 (21%)	69 (48%)
17	641 (13%)	447 (70%)	327 (51%)	468 (73%)	246 (6%)	90 (37%)	57 (23%)	101 (41%)
18	907 (18%)	593 (65%)	419 (46%)	616 (68%)	474 (10%)	127 (27%)	68 (14%)	162 (34%)
19	792 (15%)	467 (59%)	315 (40%)	522 (66%)	732 (15%)	147 (20%)	91 (12%)	183 (25%)
20	1,033 (17%)	586 (57%)	431 (42%)	566 (55%)	1,057 (20%)	165 (16%)	142 (13%)	211 (20%)
21≤	895 (13%)	482 (54%)	441 (49%)	420 (47%)	2,356 (39%)	325 (14%)	351 (15%)	327 (14%)
**Total**	5,405 (100%)	3,381 (63%)	2,523 (43%)	3,438 (69%)	5,244 (100%)	1,013 (22%)	787 (16%)	1,166 (25%)

Abbreviations: AUDIT, Alcohol Use Disorder Identification Test; ADF, average drinking frequency in past year; TDQ, typical drinking quantity (drinks/drinking day).

a Data are expressed as n (%) by weighted number.

b *p*-values were significant at the P<0.01 level using chi-square test.

*Note*: percentages may not add to 100, or to the subgroup total, due to rounding.

### Effect of the age at first drink on alcohol drinking pattern

**Figure 1:**
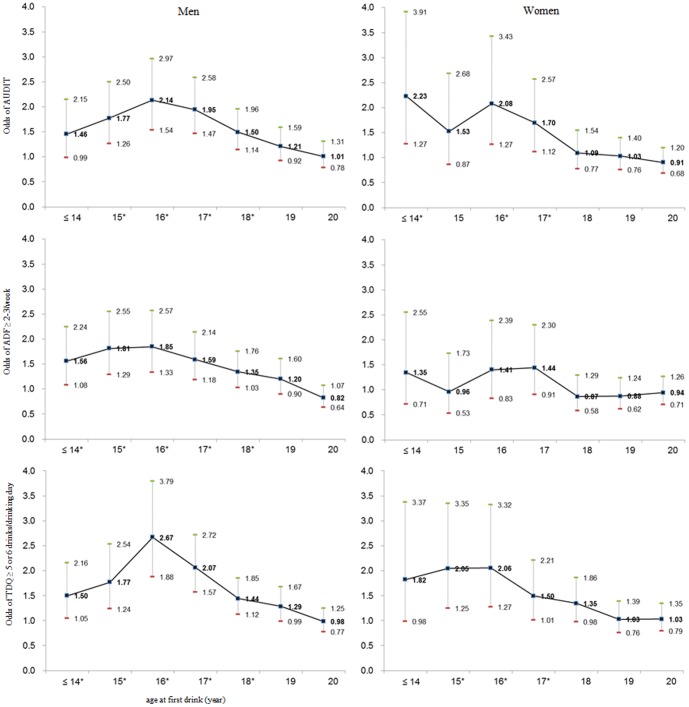
Mean adjusted odds ratios of three indicators of the adult alcohol drinking patterns from multiple logistic regressions. Abbreviations: AUDIT, Alcohol Use Disorder Identification Test; ADF, average drinking frequency in past year; TDQ, typical drinking quantity (drinks/drinking day). ^*^ *p*-values were significant at the P<0.05 level using chi-square test. *Note*: odds ratios are relative to those who started drinking at 21 years or older, controlling for age, household income, education, co-habitation, residential region, occupation, smoking, and self-rated stress level by gender. Error bars represent 95% confidence intervals.

Odds ratios and 95% CIs from multiple logistic regression analysis revealed that relative to those who waited until age 21 years or older, those who started drinking at an earlier age were more likely to have ≥8 on the AUDIT, report average drinking ≥2–3 times/week, and consume more than 5–6 drinks/drinking day (Figure 1). After controlling for age, household income, education, co-habitation, residential region, occupation, smoking, and self-rated stress level, men who first started drinking at age 14 or under had 1.46 (95% CI, 0.99–2.15) times the odds of having ≥8 on the AUDIT, 1.56 (95% CI, 1.08–2.24) times the odds of reporting average drinking ≥2–3 times/week, and 1.50 (95% CI, 1.05–2.16) times the odds of consuming more than 5–6 drinks/drinking day relative to those who waited until they were 21 years or older. For women relative to those who started drinking at age 21 or older, those who first started drinking at age 14 or under had 2.23 (95% CI, 1.27–3.91) times the odds of having ≥8 on the AUDIT, 1.35 (95% CI, 0.71–2.55) times the odds of reporting average drinking ≥2–3 times/week, and 1.82 (95% CI, 0.98–3.37) times the odds of consuming more than 5–6 drinks/drinking day, after controlling for all the mentioned confounders.

## Discussion

This study demonstrated that alcohol drinking patterns in regards to AUDIT scores, average drinking frequency, and typical drinking quantity on drinking day in later adulthood were associated with the age at first drink in Korean. Although several studies indicated that the age at first drink is associated with later hazardous alcohol consumption [15], [17], [23], it is particularly unclear whether those associations differ by gender. An important aim for many prevention programs targeting young people for alcohol-related disorders is to delay the onset of drinking as long as possible [24], [25] and many studies have recommended that the prevention of adult heavy drinking should include delaying the onset of drinking [26], [27]. Therefore, it is essential to understand differences in the onset age of drinking and alcohol drinking patterns in adulthood between men and women so to inform the construction and evaluation of gender-specific strategies to prevent risky or hazardous alcohol consumption at a later time.

Age, co-habitation, occupation, smoking, and self-rated stress level were significantly related to the drinking patterns for men, whereas education, co-habitation, smoking, and self-rated stress level were significant factors for the drinking patterns of women. Overall, men were more likely to have higher AUDIT score and reported drinking more frequently and more daily consumed alcohol quantity than women. Regardless of gender, our results showed a significant inverse association between the age at first drink and the adult alcohol drinking patterns: persons who first started drinking at earlier age are more likely to have ≥8 on the AUDIT, report average drinking ≥2–3 times/week, and consume more than 5–6 drinks/drinking day, which means individuals with at-risk drinking level [2], [5]. Those inverse associations between the age at first drink and the alcohol drinking patterns in regards to AUDIT scores, average drinking frequency, and typical drinking quantity on drinking day in later adulthood were constant except for average drinking frequency of women even after adjusting potential confounders including age, household income, education, co-habitation, residual region, occupation, smoking, and self-rated stress level (Figure 1). Men and women participants who initiated drinking prior to age 17 reported significantly more in at-risk drinking indicators than individuals who began drinking at age 20 or later which is the legal age limit in Korea. Thus the effect of the age at first drink on adult alcohol drinking patterns was similar for both genders.

The results were in accordance with several evidences from previous studies. Both men and women experiencing binge drinking in adulthood most frequently had a drinking onset of age 14 or under, which was an association that rapidly declined after age 21 [19], [22]. Significant increasing trends were observed in the rate of alcohol consumption [5], [15], [19], frequency of drinking at young ages [1], alcohol abuse and dependence in adulthood [9], [20], frequency of heavy drinking [18], and consequent binge drinking in adulthood [16] among participants who initiated drinking at earlier age. Apart from a previous study [16], there was no significant association between the age at first drink and average drinking frequency among women in present study. Only men who initiated drinking at age 18 or under were slightly more likely to report average drinking ≥2–3 times/week and the results were statistically significant. Future study is warranted to better understand the gender-specific association between the onset age of drinking and alcohol drinking patterns.

This study has several limitations that need to be taken into consideration. First, we could not fully exclude the effects of information bias because the measurement of alcohol drinking patterns along with the age at first drink was based on a self-reported questionnaire survey. However, there is an evidence that self-assessment of alcohol consumption is reliable [28]. Second, causal inferences cannot be drawn from this study result using a cross-sectional study design. Third, there may be residual or uncontrolled confounders that influence the associations. For instance, some previous studies suggested that children with particular adverse childhood experiences may be at risk for earlier drinking initiation along with a different pattern of alcohol consumption across their life span [29], suggesting that these unknown confounders might be related to our study results. In addition, a study [30] indicated that higher educational attainment is associated with increased alcohol consumption and the relationship is stronger for women than men. If education attainment was systematically varied between younger and older generation, our analytical model could not capture the residual relationship even though education level and age variables were controlled for the multiple logistic regressions. Finally, the cut-off used to define individuals with at-risk drinking level at ≥8 on the AUDIT and 5–6 drinks/drinking day may be too high and therefore inappropriate in women. We considered the cut-off points based on previous studies [5], [22] but those studies were not designed to analyze gender differences. Therefore, those variables might be related to a ceiling effect and the estimates were somehow biased.

In conclusion, the present findings in a representative Korean population demonstrate that the age at first drink is a predictor of adult alcohol drinking patterns in both genders although men report consuming more alcohol and spend more time drinking than women. Our findings suggest that delaying the initiation of drinking might be an appropriate goal for prevention efforts and that the present level for the legal age limit (18 years) for alcohol use is relevant; in some countries it is even higher. Prescott and Kendler [29] have, however, claimed that delaying the age of onset of drinking would not prevent severe alcoholism, because so many other factors are included. By delaying the onset age, at least the hazardous effects of heavy drinking in adolescence could possibly be avoided. As proposed by previous study [12], a change in general attitudes is needed to prevent an increase in problems caused by alcohol in the future. Achieving this result will require a joint effort by everyone, including parents, media, professionals, and politicians, and will necessitate a change in adult drinking culture.
